# Disruption of oxidative balance in the gut of the western honeybee *Apis mellifera* exposed to the intracellular parasite *Nosema ceranae* and to the insecticide fipronil

**DOI:** 10.1111/1751-7915.12772

**Published:** 2017-07-24

**Authors:** Laurianne Paris, Michaël Roussel, Bruno Pereira, Frédéric Delbac, Marie Diogon

**Affiliations:** ^1^ Université Clermont Auvergne CNRS LMGE F‐63000 Clermont‐Ferrand France; ^2^ Université Clermont Auvergne CHU Clermont‐Ferrand Unité de Biostatistiques DRCI F‐63000 Clermont‐Ferrand France; ^3^ Present address: Laboratoire Microorganismes: Génome et Environnement 1 Impasse Amélie Murat – TSA 60026, CS 60026 63178 Aubière Cedex France

## Abstract

The causes underlying the increased mortality of honeybee colonies remain unclear and may involve multiple stressors acting together, including both pathogens and pesticides. Previous studies suggested that infection by the gut parasite *Nosema ceranae* combined with chronic exposure to sublethal doses of the insecticide fipronil generated an increase in oxidative stress in the midgut of honeybees. To explore the impact of these two stressors on oxidative balance, we experimentally infected bees with *N. ceranae* and/or chronically exposed to fipronil at low doses for 22 days, and we measured soluble reactive oxygen species (ROS) and ROS damage by quantifying both protein and lipid oxidation in the midgut. Our results revealed a disruption of the oxidative balance, with a decrease in both the amount of ROS and ROS damage in the presence of the parasite alone. However, protein oxidation was significantly increased in the *N. ceranae*/fipronil combination, revealing an increase in oxidative damage and suggesting higher fipronil toxicity in infected bees. Furthermore, our results highlighted a temporal order in the appearance of oxidation events in the intestinal cells and revealed that all samples tended to undergo protein oxidation during ageing, regardless of treatment.

## Introduction

Honeybees play a preponderant role in crop pollination and biodiversity conservation. Unfortunately, massive mortalities of managed honeybee populations have been reported for many years, especially in Europe and North America. For instance, the number of *Apis mellifera* colonies decreased from 4.6 million in 1970 to 2.7 million in 2014 in the USA and from 21.1 million to 17.7 million in the same time in Europe (FAO, 2017), and the colony losses reached 32.4% in some apiaries in Belgium at the end of winter 2013 (Laurent *et al*., 2016). The origin of colony losses is considered multicausal, including pathogens and predators, chemical substances (pesticides, industrial pollutants), agricultural practices and climatic conditions, but no identified individual cause is sufficient to explain the global honeybee decline recorded worldwide. This led to the consideration that interactions between multiple stressors, in particular between parasites and pesticides, might play a role in colony decline (VanEngelsdorp *et al*., 2009; Neumann and Carreck, 2010; Potts *et al*., 2010; VanEngelsdorp and Meixner, 2010; Cousin *et al*., 2013; Nazzi and Pennacchio, 2014; Goulson *et al*., 2015). Indeed, honeybee colonies are exposed through their foraging activities to a variety of chemical pollutants and pathogens, and several studies have demonstrated detrimental interactions between these stressors. Most of these studies focused on the gut parasite *Nosema ceranae*, indicating that this infectious agent can sensitize honeybees to chemical stressors (Alaux *et al*., 2010; Vidau *et al*., 2011; Aufauvre *et al*., 2012, 2014; Pettis *et al*., 2012, 2013; Wu *et al*., 2012; Retschnig *et al*., 2014). Moreover, the association of stressors may have a synergistic effect, i.e. their combined effect may be stronger than the sum of their effects taken independently (Holmstrup *et al*., 2010).

The microsporidium *Nosema ceranae* is among the most common pathogen in *Apis mellifera*, with a worldwide distribution (Goulson *et al*., 2015). This obligate intracellular parasite invades and develops within the cytoplasm of the epithelial cells of the adult honeybee midgut and has been associated with the weakening of honeybee colonies (Higes *et al*., 2006; Fries, 2010). Honeybees become infected when they ingest *Nosema* spores, the faecal–oral and oral–oral (exchange by trophallaxis) routes of transmission being the main modes of transmission (Higes *et al*., 2010; Smith, 2012). Fipronil is an insecticide in the phenylpyrazole family that acts as a reversible inhibitor of the gamma‐aminobutyric acid (GABA) receptor and glutamate‐activated chloride channels. It is widely used for control of arthropods and especially lepidopteran pests, in the form of dispersible granules, concentrated aqueous solutions or seed coating, on worldwide crops pollinated by bees.

Both *Nosema ceranae* and fipronil may alter the behaviour, the physiology and the survival of honeybees at different levels. At the social level, these stressors may induce many changes in the colony organization (Antúnez *et al*., 2009; Alaux *et al*., 2010; Dussaubat *et al*., 2012; Goblirsch *et al*., 2013) and may disturb foraging activities by reducing homing and orientation abilities (Colin *et al*., 2004; Mayack and Naug, 2010; Decourtye *et al*., 2011; Dussaubat *et al*., 2013). At the individual level, fipronil induces impaired learning and memory (Decourtye *et al*., 2005; El Hassani *et al*., 2005; Aliouane *et al*., 2009; Bernadou *et al*., 2009), while *N. ceranae* can cause severe nutritional and energetic stress (Mayack and Naug, 2010; Aliferis *et al*., 2012) and a progressive and irreversible degeneration of the gut epithelium that can lead to disorders of digestive function (García‐palencia *et al*., 2010; Dussaubat *et al*., 2012). At the cellular level, infection by *N. ceranae* results in a significant decrease in the rates of host energetic resources such as ATP and carbohydrates (Higes *et al*., 2007; Williams, 2009; Mayack and Naug, 2010; Aliferis *et al*., 2012) and may also prevent apoptosis in epithelial cells of the infected midgut (Higes *et al*., 2013; Kurze *et al*., 2015; Martín‐Hernández *et al*., 2017). Finally, both *N. ceranae* and fipronil can affect the humoral response by decreasing the expression of antimicrobial peptide‐encoding genes (Antúnez *et al*., 2009; Chaimanee *et al*., 2012; Aufauvre *et al*., 2014). Interestingly, different studies have also shown that the combination of *N. ceranae* and sublethal doses of fipronil led to a significant decrease in honeybee survival (Vidau *et al*., 2011; Aufauvre *et al*., 2012, 2014).

Reactive oxygen species (ROS) are a key component of the innate immune response in insects. In *Drosophila melanogaster*, ROS‐mediated immunity was shown to be particularly important to fight pathogens in the midgut (Lemaitre and Hoffmann, 2007; Ferrandon, 2013; Buchon *et al*., 2014). These reactive molecules and free radicals derived from molecular oxygen are permanently generated by different physiological mechanisms at low‐to‐moderate concentrations as signalling mediators or defensive molecules, or during mitochondrial respiration. They can also be generated from exogenous sources such as food, pollutants or toxins (Batty *et al*., 2009; Bevilacqua *et al*., 2012; Dupré‐Crochet *et al*., 2013; Nathan and Cunningham‐Bussel, 2013). In contrast, when they are produced at high concentrations, they can damage numerous components of the cell including lipids, proteins and DNA (Kohen and Nyska, 2002). Fortunately, most organisms possess antioxidant systems to control and regulate the level of ROS (Delattre *et al*., 2005; Dupré‐Crochet *et al*., 2013). Consequently, in physiological conditions, the antioxidant/pro‐oxidant balance is in equilibrium, but in some cases, an excess of ROS can occur, either by antioxidant deficit or because of overproduction of radicals. This imbalance is referred to as oxidative stress (Delattre *et al*., 2005). Oxidative stress is transient and generally tissue specific. One methodology for determination of oxidative stress consists of direct detection of ROS and other radicals, but this remains rather difficult because these molecules are very short‐lived and highly reactive. Thus, oxidative stress may be measured through quantification of oxidative damage markers (lipid, protein and DNA oxidation) or quantification of the antioxidant defence system (Kohen and Nyska, 2002).

The honeybee genome revealed a specific lack of several genes involved in innate immunity and detoxification enzymes compared with the genomes of *Drosophila melanogaster* and *Anopheles gambiae* (Claudianos *et al*., 2006; Evans *et al*., 2006; du Rand *et al*., 2015). This deficit in genes involved in both detoxification and immunity processes is compensated for by the eusocial organization of bee colonies (Nikolenko *et al*., 2012). However, many transcriptomic studies showed that the expression of different genes involved in the antioxidant system was significantly increased in the bee midgut during infection with *N. ceranae* (Dussaubat *et al*., 2012; Aufauvre *et al*., 2014). This parasite also induced changes in the gut in both the quantity and the activity of different ROS‐scavenging enzymes (Vidau *et al*., 2011, 2014; Dussaubat *et al*., 2012). All these data suggest an increase in the production and enzymatic activity of components of the antioxidant system in response to eventual ROS production during infection by *N. ceranae*.

In the present study, our objective was to determine whether exposure to the insecticide fipronil and/or infection by *Nosema ceranae* may disturb the pro‐oxidative/antioxidative balance in the honeybee midgut, which is the target tissue of both *N. ceranae* development and fipronil absorption. In addition, we chose to compare the assumptive pro‐oxidant effect of fipronil (Ki *et al*., 2012; Carvalho *et al*., 2013) to the effect of N‐acetylcysteine, an antioxidant commonly used in oxidative stress studies (Ki *et al*., 2012; Sun *et al*., 2012; Cousin *et al*., 2013). We directly measured ROS compounds and developed techniques to quantify cell damage in honeybee intestinal cells. A primary experiment was performed to quantify the amount of ROS in the midgut of infected honeybees. A secondary experiment was then conducted to measure peroxide production and cell damage (protein and lipid oxidation) in the midgut of *Nosema*‐infected and/or fipronil‐intoxicated bees.

## Results

### Experiment 1: ROS measurement in the midgut of *Nosema ceranae*‐infected versus uninfected honeybees

**Figure 1:**
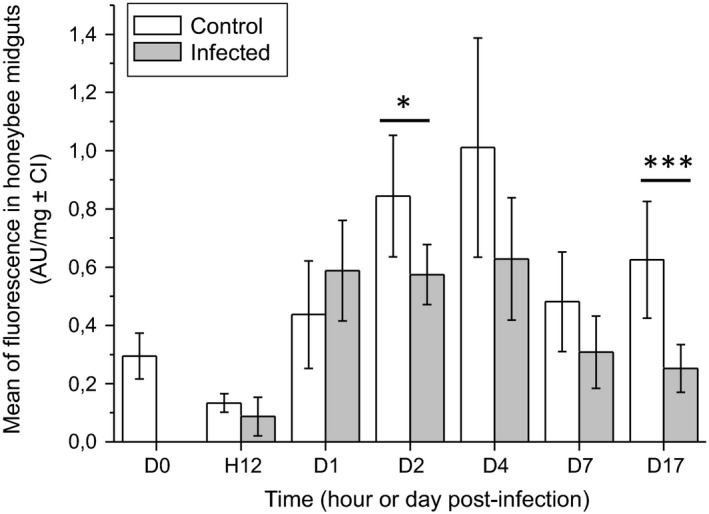
Comparison of ROS concentration between *Nosema ceranae*‐infected and uninfected honeybees. The ROS concentration was measured in uninfected (control) and infected honeybee midguts using the ROS‐sensitive CM‐H_2_ DCFDA fluorescent probe from D0 (time of infection, 5 days after emergence of bees) to D17. The fluorescence was calculated by deducting the natural fluorescence (mean fluorescence of midgut incubated with the probe subtracted from mean fluorescence of midgut unincubated with the probe) from the total fluorescence. Asterisks indicate significant differences between control and infected groups, calculated with a Mann–Whitney *U*‐test (*P ≤ 0.05; ***P ≤ 0.001).

To evaluate the global oxidative status of the *Apis mellifera* midgut upon infection by *Nosema ceranae*, a ROS‐sensitive fluorescent probe (CM‐H_2_DCFDA) was used to detect and quantify reactive oxygen species (ROS) in both uninfected and infected bees at different hours (H) or days (D) post‐infection, from D0 to D17. Statistical analyses did not show any difference between control (i.e. uninfected) and infected honeybees except on 2 days, D2 (*P* = 0.043) and D17 (*P* = 0.001). On those days, the amount of ROS significantly decreased in infected bee midguts compared with those of the control group (Fig. 1).

### Experiment 2: analysis of the oxidative balance in honeybee midguts following infection by *Nosema ceranae* and/or exposure to the insecticide fipronil, or the N‐acetylcysteine antioxidant

In this experiment, six experimental groups were created: (i) untreated control (Control), (ii) infected with *N. ceranae* (Infected), (iii) uninfected and fed with 1 mM of the antioxidant N‐acetylcysteine (NAC), (iv) infected and fed with N‐acetylcysteine (INAC), (v) uninfected and chronically exposed to 0.5 μg l^−1^ of fipronil (FIP) and (vi) infected and chronically exposed to fipronil (IFIP). Each group was monitored daily (from D0 to D22) to evaluate their survival rate and consumption behaviour. To further analyse the oxidative balance, honeybee midguts were collected to track the production of soluble peroxides as well as the damage potentially generated by ROS (oxidation of lipids and proteins).

### Survival analysis and consumption behaviour

**Figure 2:**
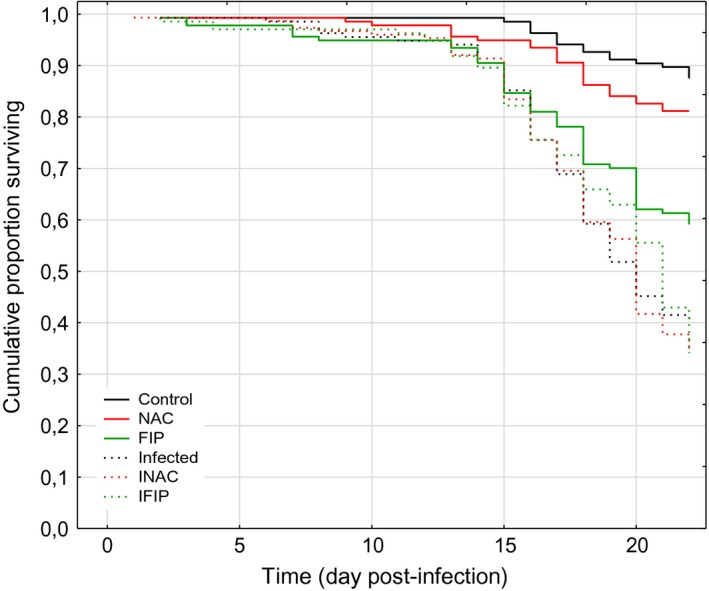
Survival analysis of honeybees after infection by *Nosema ceranae* and/or exposure to the insecticide fipronil or to the antioxidant N‐acetylcysteine.The data show the cumulative proportion of surviving honeybees exposed to no treatment (Control), to *N. ceranae* (Infected), to fipronil 0.5 μg l^−1^ (FIP), to N‐acetylcysteine 1 mM (NAC), to *N. ceranae*–fipronil combination (IFIP) or to a combination of *N. ceranae* and NAC (INAC). The data from three replicates of 45 bees for each experimental condition were analysed with the Kaplan–Meier method and the Cox–Mantel test (see Table S1). D0 corresponded to the day of honeybee infection, 5 days after their emergence.

Survival analysis indicated that untreated bees (Control) and bees fed with the antioxidant N‐acetylcysteine (NAC) had the lowest mortality rate (< 20%) at the end of the experiment, i.e. D22 (Fig. 2). In contrast, when bees were chronically exposed to the insecticide fipronil (FIP), the mortality rate significantly increased and reached approximately 40% at D22 (*P* < 0.001). All three *N. ceranae*‐infected groups (Infected, INAC and IFIP) also showed a significant decrease in bee survival (*P* < 0.001) compared with their uninfected counterparts (Control versus Infected; NAC versus INAC; FIP versus IFIP; *P* < 0.001), but there was no significant difference in the survival probability among the three infected groups (see Table S1).

**Figure 3:**
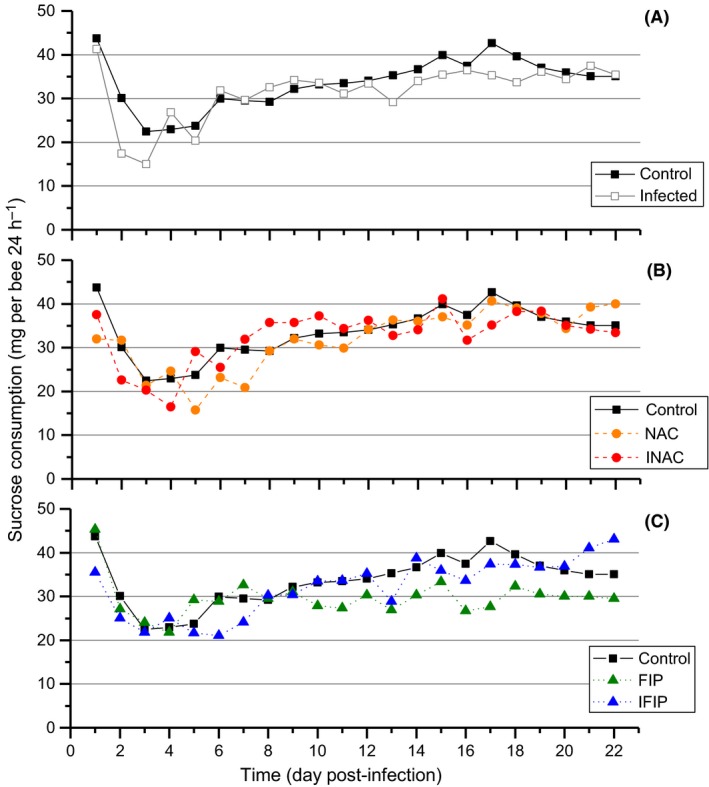
Daily sucrose consumption curves of honeybees for the six experimental groups. The data represent the mean sucrose consumption for three cages per condition (mg per bee per 24 h) monitored daily from D0 to D22 in the three uninfected groups (Control, NAC, FIP) and the three infected groups (Infected, INAC, IFIP). The quantity of sucrose consumed was measured daily by weighing the feed tubes. A mixed model analysis was performed to compare dietary behaviour in the six different conditions (see Table S1). The control was compared to the infected group (A), NAC and INAC groups (B) and FIP and IFIP groups (C). D0 corresponded to the day of honeybee infection, 5 days after their emergence.

The monitoring of daily sucrose consumption revealed a sudden drop in consumption (fold change of approximately 1.8 ± 0.5) during the 4 days after anaesthesia with CO_2_ in all six experimental groups (Fig. 3). Mixed model statistical analysis (Table S1) of daily consumption indicated a similar consumption profile for bees exposed to NAC (NAC), to *N. ceranae* (Infected) and to the *N. ceranae*–fipronil combination (IFIP) compared with the control group. In contrast, the daily sucrose consumption stopped increasing for groups exposed to fipronil alone (FIP) and to the *N. ceranae*–NAC combination (INAC) from days D5 and D7, respectively, and the mean stabilized at 30 ± 5 and 35 ± 5 mg bee^−1^ day^−1^ respectively. For all combined days, the comparison among the six groups highlighted a different level of consumption for the fipronil‐exposed (FIP) group, with a 1.14‐fold decrease in sucrose consumption compared with the control group (*P* < 0.001). Fipronil‐exposed honeybees absorbed an average of 0.013 ng of fipronil per bee per day; similarly, bees co‐exposed to *N. ceranae* and fipronil (IFIP) consumed an average of 0.014 ng bee^−1^ day^−1^. The oral LD_50_ of fipronil is 4.2 ng bee^−1^, and thus, we determined that the bees surviving at the end of experiment (D22) had received 1/14.6 and 1/13.5 of the LD_50_ for the FIP and IFIP groups respectively. It can also be noted that similar quantities of the antioxidant had been ingested by uninfected (NAC, 5.2 μg bee^−1^ day^−1^) and infected (INAC, 5.3 μg bee^−1^ day^−1^) bees (see Table S1).

### Nosema ceranae development

**Figure 4:**
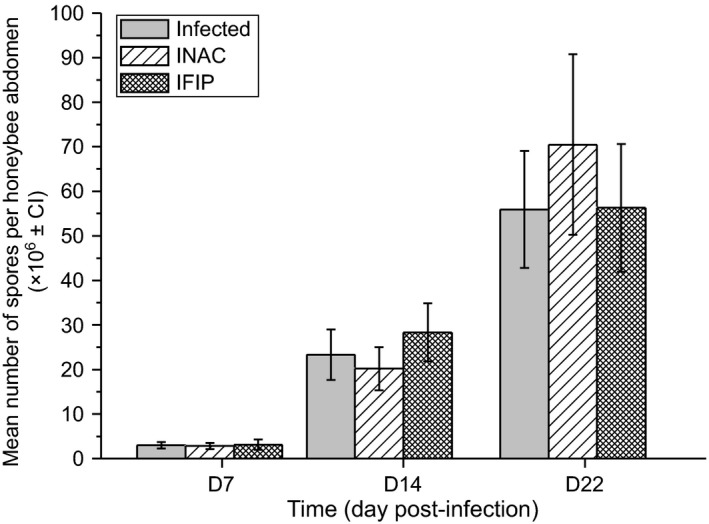
Effect of exposure to fipronil or N‐acetylcysteine on *N. ceranae* spore production.The spore production in the whole abdomen was evaluated at 7, 14 and 22 days post‐infection for the three conditions of infection (*N. ceranae* alone (Infected); supplemented with N‐acetylcysteine 1 mM (INAC) or fipronil 0.5 μg l^−1^ (IFIP)). The data represent the mean number of spores per honeybee abdomen (×10^6^; error bars represents 95% confidence interval) from 24 (8 × 3 cages) honeybees per condition for D7 and D14 and 15 honeybees (5 × 3 cages) per condition for D22. A Kruskal–Wallis test followed by Dunn's test was applied to compare the parasite load in the three infected groups (see Table S1).

The success of *N. ceranae* infection was monitored by counting the spores present in the whole bee digestive tract at 7, 14 and 22 days post‐infection. No significant difference was observed among the three infected groups (Infected, INAC and IFIP; Fig. 4). The absence of *N. ceranae* in the control groups was confirmed by examining the contents of the abdomens by microscopy.

### Quantification of soluble peroxides, lipid peroxidation and protein carbonylation in honeybee midguts

To evaluate the oxidative status of midguts under infection by *N. ceranae* and/or exposure to fipronil or N‐acetylcysteine, we quantified the amount of soluble peroxides and measured oxidative damage by quantifying both lipid peroxidation and protein carbonylation.

#### Determination of oxidative events

**Figure 5:**
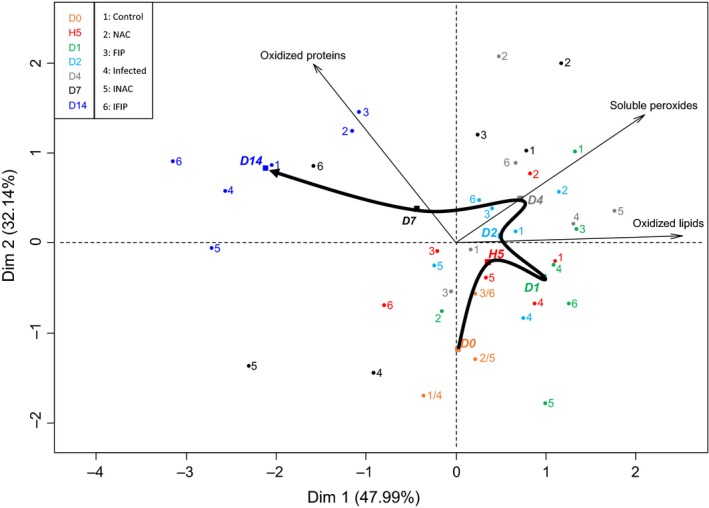
Principal component analysis of the three variables (lipid oxidation, soluble peroxide production and protein oxidation) in the honeybee midguts during the experiment timeline. The points of each group at different times in the experiment (D0–D14) were projected, and the barycentre of each day for all combined conditions was determined. The bold black arrow connects barycentre points to highlight the temporal order of the three events: lipid oxidation, soluble peroxide production and protein oxidation.

Principal component analysis (PCA) revealed relationships between three initial variables, underlying an opposite relation between oxidized lipids and oxidized proteins (Fig. 5). Furthermore, inertia of soluble peroxide points was shared between two dimensions and, consequently, between oxidized lipids and proteins. The correlation between oxidized lipids and soluble peroxides was not significant. Figure 5 depicts a timeline of the appearance of experimental effects on oxidative balance, considering the barycentre of all the conditions for each day observed. Indeed, it seemed that lipids underwent oxidation over time before an increase of soluble peroxides in the midgut. Then, at D14, all samples tended towards protein oxidation. We also noted that the samples were well grouped at D0 (just before infection). Between H5 and D7, the sample points of the PCA seemed more scattered, reflecting different behaviour of samples depending on treatments, but they were all gathered at D14 and tended towards protein carbonylation.

#### Oxidative damages and soluble peroxide production

**Figure 6:**
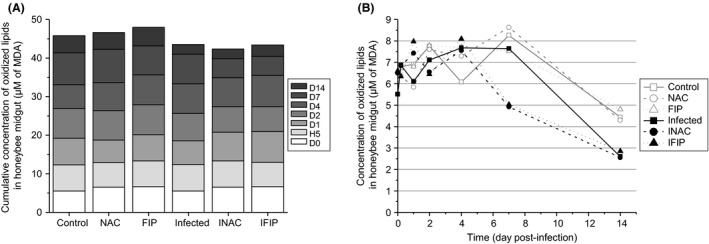
Lipid peroxidation quantification in the midguts for the six experimental groups.The amount of oxidized lipids was determined from nine pools of three midguts for each of the six conditions per day, at seven time points, from D0 to D14. A represents the cumulative concentration of oxidized lipids of each experimental group, and B represents the kinetics of the oxidized lipid concentrations for the six groups. Each experimental group was compared with the control. Details regarding differences and *P*‐values are reported in supporting information Tables S2 and S3.

Mixed model analysis was used individually for the three experiments (soluble peroxides, lipid peroxidation and protein carbonylation). Concerning the lipid peroxidation, no significant difference was observed among all groups compared with the untreated group (Control) when we considered all combined days (Fig. 6A). Nonetheless, we observed a global slight increase in lipid oxidation in the fipronil‐treated group (FIP) compared with the control group and a downward trend in the three infected groups (Infected, INAC and IFIP). In the infection–fipronil combination (IFIP), we observed a 1.2‐fold decrease in lipid oxidation compared with bees only exposed to fipronil (FIP; Fig. 6A). Figure 6B shows the kinetic of lipid peroxidation and highlights an important decrease in lipid oxidation for each group at D14. At this time, the average concentration of oxidized lipids was 4.5 ± 0.3 μM in the three uninfected groups (Control, NAC and FIP), while it was only 2.7 ± 0.2 μM in the three infected groups (Infected, INAC and IFIP). These differences were statistically confirmed (see Table S3). Interestingly, this decrease occurred earlier (from the 4th day post‐infection) in both the INAC and IFIP groups compared with the other groups. However, in contrast to the next experimental measures (protein carbonylation and soluble peroxide quantification), the lipid peroxidation could not be determined at D22 due to the low bee survival on this day.

**Figure 7:**
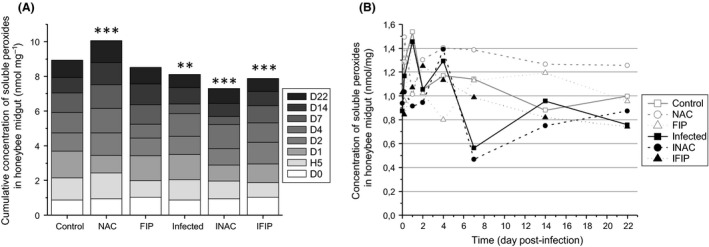
Concentration of soluble peroxides in the midguts of honeybees exposed to fipronil or N‐acetylcysteine and/or infected with *N. ceranae*.The amount of peroxides was evaluated with the ferrous ion oxidation–xylenol orange (FOX) method from 21 midguts/day/condition in quadruplicate. A represents the cumulative concentration of soluble peroxides for each group, and B represents the kinetics of the soluble peroxide concentrations for each group from D0 to D22. Asterisks indicate significant differences compared with the control group (**P ≤ 0.01; ***P ≤ 0.001). Details regarding *P*‐values can be consulted in supporting information Tables S2 and S3.

We also measured the amount of soluble peroxides at eight time points from D0 to D22. Statistical analyses showed that the antioxidant treatment (NAC) led to a global increase in soluble peroxides in midguts compared with untreated bees (Control). In contrast, the cumulative concentration of soluble peroxides was significantly reduced in the three infected groups (Infected, INAC and IFIP) compared with the control group and was lowered compared with their respective controls (Control versus Infected; NAC versus INAC; FIP versus IFIP; *P* < 0.01; Fig. 7A and Table S2). Figure 7B revealed the kinetics of soluble peroxide production between the different groups with reference to the control group. We could observe in the control group a production peak at 1.54 nmol mg^−1^ on D1, followed by stable production of approximately 1.05 ± 0.11 nmol of peroxides per mg of midgut between D2 and D22. In uninfected bees exposed to the antioxidant (NAC), this peak of soluble peroxides appeared earlier, at H5 (1.49 nmol mg^−1^), and the concentration remained stable from D2 to D22 (1.32 ± 0.07 nmol mg^−1^) but was 1.3‐fold higher than that found in the untreated control. Bees only exposed to fipronil (FIP) presented less regular kinetics compared with the control group, but no difference could be observed when all combined days were considered (see Fig. 7A). The *N. ceranae*‐infected group (Infected) presented the same kinetics as the control group except for a significant decrease at D7, similar to what was observed in the INAC group, at approximately 0.50 ± 0.07 nmol mg^−1^ after a previous peak at D4. Contrary to other groups, the INAC group was relatively stable during the two‐first days post‐infection (mean of 0.96 ± 0.05 nmol mg^−1^ from D0 to D2). Finally, the group of bees exposed to the *N. ceranae*–fipronil combination (IFIP) presented unique kinetics compared with the other infected groups, with a progressive 1.7‐fold decrease between D2 and D22. At the end of the experiment (D22), the concentration of soluble peroxides was significantly higher in the three uninfected groups than in their respective infected groups (Control versus Infected; NAC versus INAC; FIP versus IFIP; see Table S3).

**Figure 8:**
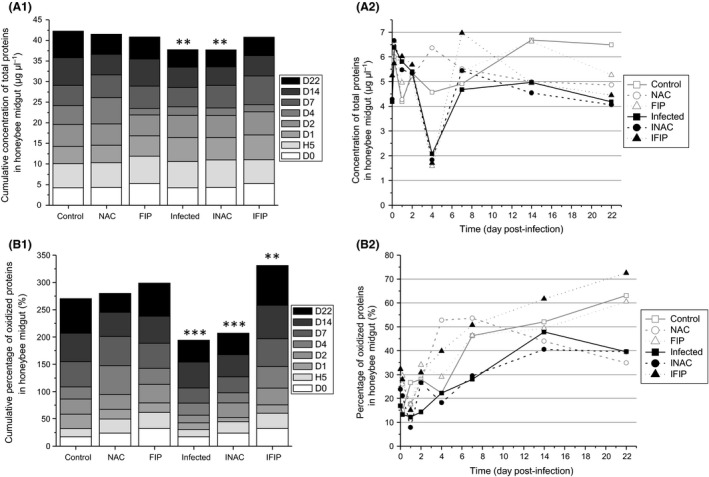
Effect of the treatments on both protein concentration and protein oxidation. The measures were performed from 12 midguts/day/condition.A. Quantification of midgut total protein concentration: A1, cumulative protein concentration for each group. A2, kinetic of protein concentration for each group.B. B1, cumulative percentage of oxidized protein for each group. B2, kinetic of percentage of oxidized protein in bee midguts for the six experimental groups. Asterisks in A1 and B1 indicate significant differences compared with the control group (**P ≤ 0.01; ***P ≤ 0.001). Details regarding *P*‐values are reported in supporting information Tables S2 and S3.

To normalize the quantification of oxidized proteins, we first measured the concentration of total proteins in the bee midguts. Statistical analysis showed a decrease in protein concentration in the three infected groups (Infected, INAC and IFIP), which was only significant in the Infected and INAC groups. In these two groups, the cumulative protein concentration decreased approximately 1.4‐fold compared with the control group (Fig.** 8A1 and Table S2). Kinetic analysis of protein concentrations from D0 to D22 revealed that major differences between groups occurred at day 4 (Fig. 8A2). At D22, the three infected groups exhibited a significant decrease of approximately 1.5‐fold compared with the control. The percentage of protein oxidation was then measured for each experimental group using a slot blot method after loading with the same concentration of total proteins. Figure 8B1 shows the cumulative percentage of protein oxidation between the different treatments. The Infected and INAC groups presented a major decrease of 1.4‐ and 1.3‐fold, respectively, compared with the control. Surprisingly, a slight but not significant increase was observed in FIP compared with the control group. This trend was accentuated in the presence of *N. ceranae* (IFIP), with an increase of 1.2‐fold compared with the control group and 1.7‐fold compared with the infected group. The kinetics of protein oxidation from D0 to D22 is presented in Fig. 8B2. Except for the NAC group, we noted a constant increase in protein oxidation over time (highlighted with the PCA; see Fig. 5), which was more important and more constant in the IFIP group.

## Discussion

The paradox of oxidative balance is that free radicals are extremely dangerous species, able to cause several types of cell damage, while being essential to life. Reactive oxygen species (ROS) serve important cell functions including signal transduction (Finkel, 2011), cell cycle regulation (Chiu and Dawes, 2012) and immune defence against pathogens (Dupré‐Crochet *et al*., 2013). They also play some roles in cell differentiation, apoptosis (Delattre *et al*., 2005) and gene regulation (redox control genes). Free radicals and ROS act as intra‐ and extracellular messengers, and their production may induce cellular responses to many stressors such as ultraviolet radiation, industrial pollutants and pesticides (Nathan and Cunningham‐Bussel, 2013). At high concentrations, ROS can cause damage to different cell components such as DNA, lipids and proteins. For all these reasons, the organism needs to control the level of ROS in order to prevent oxidative stress, i.e. a disturbance in the balance between the production of ROS and antioxidants.

In our study, we were wondering whether infection with the parasite *N. ceranae* combined with exposure to low doses of the insecticide fipronil could induce a disturbance of the oxidative balance in the honeybee midgut. Most of the studies conducted on ROS production in *N. ceranae*‐infected bees focused on the detoxification system for these molecules, either through analyses of the midgut transcriptome or *via* measurement of the activity of enzymes involved in antioxidant systems (Vidau *et al*., 2011; Dussaubat *et al*., 2012; Aufauvre *et al*., 2014). These studies suggested an increase in ROS production during infection by *N. ceranae*, but no direct ROS quantification had been performed. In addition, exposition of bees to fipronil at low doses (1/10 and 1/20 of LD_50_) led to an increase of catalase activity in the midgut, suggesting that fipronil could induce oxidative stress by producing H_2_O_2_ (Carvalho *et al*., 2013). It should be noted that ROS quantification is challenging because of the short lifespan of these molecules and their high reactivity (nanoseconds to seconds according to the molecular species).

We decided to measure soluble peroxides and ROS‐induced damages through the quantification of both protein and lipid oxidation in the gut. The choice of the administered fipronil dose was based on preliminary tests and on the oral LD_50_ value of 4.17 ng bee^−1^ (Kievits and Bruneau, 2007). Consequently, bees were exposed daily to a mean quantity of fipronil of 1/298 of the LD_50_. Considering the total duration of the experiment (22 days), the honeybees received a cumulative quantity of fipronil equivalent to the LD_50_/14. Although this dose was initially considered sublethal (Vidau *et al*., 2011), a significant mortality rate was observed in the fipronil‐exposed group (approximately 40%) compared with the control group from the 15th day after the chronic exposure. We analysed the mortality rate and the feeding behaviour of honeybees without considering only cumulative consumption, commonly used in some studies (Mayack and Naug, 2009; Vidau *et al*., 2011), because this method may fail to reflect daily differences induced by the different treatments. The combined monitoring of mortality and sucrose consumption revealed that the increased mortality of bees co‐exposed to *N. ceranae* and fipronil (IFIP) versus FIP group was not the result of more ingestion of fipronil. Indeed, despite a different consumption profile between FIP and IFIP, the total quantities absorbed were similar between these two groups (mean of 0.01 ng bee^−1^ day^−1^). In contrast, a difference has been found in the daily consumption between bees fed fipronil (FIP group) and the untreated control group (*P* < 0.01; Fig. 3). This difference may suggest that fipronil consumption negatively affected the appetite of the bees or that fipronil made the syrup less appealing to bees. However, no difference has been found in the daily consumption profile between the IFIP group and the control group. We thus hypothesized that the presence of the parasite may hide the negative effect of fipronil on consumption behaviour.

Our data indicated that infection with *N. ceranae* had a more severe impact on honeybee survival than exposure to low doses of fipronil. At the end of the experiment (D22), mortality reached approximately 60% in the group of *N. ceranae*‐infected bees and approximately 40% for bees exposed to fipronil alone. Surprisingly, and in contrast to other studies (Vidau *et al*., 2011; Aufauvre *et al*., 2012), the *N. ceranae*–fipronil combination did not induce higher honeybee mortality compared with the infected group (Fig. 2). Additionally, the administration of the antioxidant N‐acetylcysteine to both uninfected (NAC group) and infected bees (INAC group) did not modify the mortality rate compared with the control and infected groups, respectively. Both *N. ceranae*‐infected and uninfected bees fed N‐acetylcysteine consumed similar amounts of the antioxidant (approximately 5.25 ± 0.05 μg bee^−1^ day^−1^). Several studies revealed differences in honeybee susceptibility to infection by *N. ceranae*. Such differences may result from resistance or tolerance mechanisms mediated by the genetic variability of honeybees (Fontbonne *et al*., 2013; Kurze *et al*., 2016a,b) but could also be due to life‐history traits of bee colonies used in the different studies. Indeed, sampled bees used for experimental studies may have been impacted in their colony by multiple factors including resources, contaminants or pathogens. All these factors may also influence the susceptibility of bees to fipronil in our laboratory conditions. Altogether, this could explain the variability of honeybee mortality rates observed in different studies under exposure to the *N. ceranae*–fipronil combination (Vidau *et al*., 2011; Aufauvre *et al*., 2012, 2014). In these studies, the *N. ceranae*–fipronil combination led to an increase in honeybee mortality with or without a synergistic effect, while in the present study, the mortality rate was similar between bees treated with a single stressor and those exposed to a combination.

Ingestion of fipronil by honeybees did not have any effect on the *N. ceranae* spore production at the different time points (D7, D14 and D22) of the experiment (Fig. 4). Similar results have been previously reported by Aufauvre *et al*. (2012). In contrast, Vidau *et al*. (2011) observed significantly decreased spore production in infected bees exposed to fipronil compared with bees only infected with *N. ceranae*. However, in the latter study, exposure to another insecticide, thiacloprid, increased the *Nosema* spore count. Such contrasting effects of pesticides including insecticides, acaricides, herbicides and fungicides on *Nosema* spore load have been reported by Pettis *et al*. (2013) and Collison *et al*. (2016). Our study also revealed that the addition of N‐acetylcysteine did not affect the spore load and did not increase survival, suggesting that this antioxidant neither stimulated the defences of *A. mellifera* against *N. ceranae* nor supported the parasite proliferation.

Oxidative stress is defined as a disturbance in the oxidative balance between the production of ROS and antioxidant defence molecules. Oxidative balance is required to preserve cellular homeostasis and to regulate ROS levels to enable their beneficial effects in many cell functions while preventing their toxic effects (Wang *et al*., 2013). When honeybees were infected with *N. ceranae* (whatever the infected group, Infected, INAC or IFIP), the balance was disturbed in favour of a decrease in soluble peroxides (Fig. 7) and oxidized proteins (Fig. 8B). In contrast, when all days were observed, lipid oxidation (Fig. 6A) did not seem to be affected by the infection. However, on the 14th day, a significant decrease in lipid oxidation was observed in the three infected groups compared with the three uninfected groups (Fig. 6B and Table S3). As mortality was high in the three infected groups, the measurement of lipid oxidation could not be made after D14, while the quantification of both soluble peroxides and oxidized proteins was performed until D22. For this reason, we cannot exclude the possibility that lipid peroxidation could be affected later (i.e. after D14). Several studies have shown an increase in the activation of the detoxification system in honeybees infected with *N. ceranae*. Indeed, we observed a global increase in the expression of genes involved in detoxification such as those coding catalase, cytochrome P450 enzymes and glutathione peroxidase (Dussaubat *et al*., 2012; Aufauvre *et al*., 2014). However, at the enzymatic level, only glutathione‐S‐transferase (GST) exhibited higher activity in infected bees (Dussaubat *et al*., 2012). GST activity was also shown to significantly increase in both the fat body and the midgut of adult workers 10 days after infection with *N. ceranae* (Vidau *et al*., 2011). Furthermore, a proteomic analysis revealed that some antioxidant proteins including thioredoxin‐like 2 and GST were more abundant in the midgut of infected honeybees (Vidau *et al*., 2014). This increase in the detoxification system is consistent with our results as ROS markers (soluble peroxides and protein carbonylation) were decreased in *N. ceranae*‐infected bees compared with uninfected bees. Interestingly, a decrease in ROS in the haemolymph of *Galleria mellonella* larvae was observed when they were infected with the microsporidium *Vairimorpha ephestiae* (Lozinskaia *et al*., 2004). This decrease seemed to be linked to a decrease in phenoloxidase activity during the infection and to an increase in SOD and GST activity during the massive sporulation of *V. ephestiae*. Altogether, this ROS decrease could be the result of a manipulation by the parasite to protect itself from its host or a counterbalancing effect of increased energetic costs in the host due the host‐derived energy dependence of microsporidia. Indeed, microsporidia possess remnant mitochondria referred to as mitosomes that do not produce ATP. For this reason, these parasites are highly dependent on host mitochondria for ATP uptake, and during proliferation, they recruit host mitochondria near their plasma membrane (Higes *et al*., 2007; Tsaousis *et al*., 2008; Hacker *et al*., 2014). However, oxidative phosphorylation generates not only ATP but also ROS from complexes I and III (Batty *et al*., 2009; Finkel, 2011). Therefore, it can be hypothesized that *N. ceranae*, by increasing mitochondrial activity, may induce an augmentation of ROS production, thus triggering an increase in the detoxification systems of the host to counteract them. We also showed that *N. ceranae* infection led to a decrease in protein concentration in honeybee midguts (Fig. 8A). This is consistent with a recent study indicating that *N. ceranae* may affect hypopharyngeal gland protein synthesis, supporting the assertion that *N. ceranae* may disrupt protein metabolism in bees (Jack *et al*., 2016). An elevation of peroxide concentration in midguts might indicate the activity of Duox, an NADPH oxidase known to be involved in gut immunity against several pathogens in *Drosophila* (Lemaitre and Hoffmann, 2007; Ferrandon, 2013; Buchon *et al*., 2014). In our study, the decrease in soluble peroxides observed in all infected groups suggests that NADPH oxidase do not seem to be activated in the gut in the presence of *N. ceranae*.

Surprisingly, a significant increase in soluble peroxides was observed in uninfected bees fed N‐acetylcysteine (NAC; Fig. 7), suggesting that the oxidative system is unbalanced in favour of peroxide production in the presence of the antioxidant. In contrast, in *Nosema*‐infected bees treated with N‐acetylcysteine (INAC), soluble peroxides, protein carbonylation and lipid peroxidation were decreased compared with the untreated control group. In addition, the decrease in the amount of soluble peroxides was significantly higher in infected bees treated with N‐acetylcysteine (INAC) than in bees only infected with *N. ceranae*. This suggests that the antioxidant may strengthen the effect of *N. ceranae* infection on soluble peroxide production.

Fipronil may act as a pro‐oxidant molecule in neuronal cell cultures when used at 50–100 μM (Ki *et al*., 2012) and in bee midguts when it is assayed at 1/10 or 1/20 of the LD_50_ (Carvalho *et al*., 2013). In our study, although honeybees absorbed a mean quantity of fipronil of 1/14 of the LD_50_, we did not observe any significant difference in the quantity of soluble peroxides or oxidative damage markers (lipid and protein oxidation) in uninfected bees treated with fipronil compared with the untreated control. In contrast, protein carbonylation increased 1.2‐fold in the infected fipronil group (IFIP) compared with the control and by 1.7‐ and 1.6‐fold compared with the Infected and INAC groups respectively. Protein carbonylation is an irreversible post‐translational modification that can strongly alter protein function, leading to cell damage (Dalle‐Donne *et al*., 2006). Proteins thus modified cannot be repaired and must be eliminated by the cell (Costa *et al*., 2007); accordingly, carbonylated proteins are considered stable and suitable markers for oxidative stress that can be induced by almost all types of ROS (Shacter, 2000). Our results suggest that the pro‐oxidant effect of fipronil would be only observed in bees infected with *N. ceranae*. This indicates that co‐exposure of bees to both *N. ceranae* and fipronil would lead to increased oxidative stress. Similarly, increased oxidative stress was described in gammarids infected with the microsporidium *Dictyocoela* and exposed to cadmium, suggesting that infected organisms would be more susceptible to pollutants (Gismondi *et al*., 2012).

As shown by the principal component analysis, no direct correlation could be observed between the production of soluble peroxides, lipid peroxidation and protein carbonylation. Although we hypothesized that production of soluble peroxides could cause direct damage to lipids and proteins, our results highlighted a temporal order in the appearance of the three oxidative stress markers upon intoxication with fipronil and/or infection with *N. ceranae*. The principal component analysis has clearly demonstrated that the level of protein oxidation was independent of the levels of soluble peroxide production and lipid oxidation, because the arrows of these last two variables were not projected in the same quarter as the oxidized protein arrow. A statistical correlation test was also performed on the production of soluble peroxides and oxidized lipids, and it did not show any link between these two events. The oxidation of lipids therefore did not appear to induce the production of soluble peroxides. In our study, we also observed a tendency towards protein carbonylation at D14 for all samples, regardless of the treatment (Fig. 5). Although the amount of oxidized proteins is known to increase with age (Höhn *et al*., 2013), we hypothesized that this increase observed in bees is partly related to the decrease in vitellogenin concentration over time. Vitellogenin, which plays a predominant role in bee lifespan, is a well‐known antioxidant protein. Indeed, Seehuus *et al*. (2006) have shown that the resistance of bees to oxidative stress was linked to the expression of vitellogenin as this protein is a preferred target of carbonylation. Therefore, the observed increase in carbonylated proteins in all groups over the course of our experiment might be due to the decline in vitellogenin during the bee lifespan.

Our result, combined with other published transcriptomic and proteomic data, highlighted a decrease in the oxidative status of intestinal cells after infection with *N. ceranae*. In contrast, the higher level of protein oxidation observed for the *N. ceranae*–fipronil combination suggests that the parasite may increase fipronil toxicity. This finding could partially explain why, in some cases, we could observe a synergistic effect on *A. mellifera* mortality when *N. ceranae* was associated with low doses of fipronil.

## Experimental procedure

### Honeybee artificial rearing and infection procedure

The two experiments (Exp. 1 and Exp. 2) were performed with emergent honeybees from *Apis mellifera* (Buckfast) colonies from the same apiary at the Laboratoire Microorganismes, Génome et Environnement (UMR 6023, Université Clermont Auvergne, Clermont‐Ferrand, France). One frame of sealed brood for Exp. 1 and five frames for Exp. 2 were collected from five colonies and placed in incubators in the dark at 33°C with approximately 60% relative humidity. Emerging honeybees were collected directly on the frames, confined to Pain‐type cages and maintained in incubators for 5 days before infection with *N. ceranae*. During this time, the bees were fed *ad libitum* with 50% sucrose syrup complemented with 1% nutritional supplement (Provita’ Bee, ATZ Dietetic). To mimic the hive environment, a small piece of PseudoQueen^®^ (Contech Enterprises, Inc., Victoria, British Columbia, Canada) releasing a queen's mandibular pheromone was positioned in each cage. After 5 days of feeding, experimental groups were established and the infection procedure was performed.

*Nosema ceranae* spores were obtained according to Roussel *et al*. (2015) one month before the experimentation. Spores were counted on a Kova^®^ slide and stored at room temperature. The *N. ceranae* species identification was confirmed by PCR using specific primers amplifying the 16S rDNA gene of *N. ceranae* (NcD1 5′‐CGACGATGTGATATGGAAAATATTAA‐3′; NcR1 5′‐TCATTCTCAAACAAAAAACCGTTC‐3′) as previously described by Martín‐Hernández *et al*. (2007). PCR was performed using the ProFlex™ 96‐well PCR System (Applied Biosystems™, Thermo Fisher Scientific, Waltham, MA, USA). The PCR was performed in 50 μl of reaction mixture using the GoTaq^®^ G2 FlexiDNA Polymerase Kit (Promega, Madison, WI, USA) in the presence of 1.25 mM MgCl_2_, 0.2 mM dNTP, 0.2 μM of each primer and 0.025 U μl^−1^ DNA polymerase. After an initial DNA denaturation step at 95°C for 5 min, 35 cycles (denaturation at 95°C for 30 s, hybridization at 54°C for 30 s and elongation at 72°C for 1 min) were run, followed by a final extension step at 72°C for 15 min. Five days after their emergence, cage‐confined bees (one group for Exp. 1 and three groups for Exp. 2) were anaesthetized with CO_2_, and the cages were placed on ice before infection in order to keep the bees asleep and to inoculate the parasite easily but also to reduce the risk of injury to the bees and/or to the manipulator. Each honeybee individually received a dose of 100 000 *N. ceranae* spores in 3 μl of sucrose 50%. The uninfected bees (one group for Exp. 1 and three groups for Exp. 2) were only anaesthetized with CO_2_ and placed in cages on ice to induce the same major stress. The time of infection with *N. ceranae* determined the D0 of each experiment.

### Primary experiment (Exp. 1)

#### Experimental design

A first experiment was conducted to measure the difference in the amount of ROS in the midgut of uninfected and infected honeybees. Towards that aim, 840 bees comprising 20 individuals per cage from one frame were used to form two groups: uninfected bees (Control) and infected bees (Infected). Five days after emergence, which determined the D0 of the experiment, 420 bees received individually 100 000 spores of *Nosema ceranae*. At different times post‐infection (D0, H5, D1, D2, D4, D7 and D17), eight midguts/cage in three cages/condition (total 24 midguts/condition) were sampled. Only the first two‐thirds of the midgut were used to measure the concentration of ROS fluorescence.

#### ROS quantification with a ROS‐sensitive fluorescent probe (CM‐H_2_DCFDA)

The following protocol was developed for the first time in this experiment for the quantification of ROS in whole tissue according to the instructions of the manufacturer (Thermo Fisher Scientific). Four midguts from each cage were individually treated with the ROS‐sensitive fluorescent probe CM‐H_2_DCFDA (Molecular Probes^®^, Life Technologies, Thermo Fisher Scientific, Waltham, MA, USA), which is an indicator for different ROS (H_2_O_2_, HO, HOCl and COO·), and four other midguts per cage were not treated with the probe to determine and subtract the natural fluorescence. The probe was used at a 10 μM concentration in PBS in the presence of a catalase inhibitor at 2 mg ml^−1^ (3‐amino‐1,2,4‐triazol, Sigma‐Aldrich^®^, Saint‐Louis, Missouri, USA), and the midguts were incubated for 20 min at RT in the dark. The midguts were then washed in PBS and crushed in 100 μl of PBS. Four hundred microlitres of PBS was added, and the mixture was homogenized by vortexing. One hundred microlitres of each sample was loaded on a 96‐well plate in quadruplicate, and the plate was done read (excitation: 485 nm per emission: 538 nm). The fluorescence for one condition was calculated by deducting the natural fluorescence (fluorescence mean of midguts not incubated in CM‐H_2_DCFDA fluorescent probe), and the results from each day were compared.

### Second experiment (Exp. 2)

#### Experimental design

To obtain enough emergent bees (≈ 6500), we took five frames of sealed brood from five colonies in the same apiary. After 5 days of feeding as described in Exp. 1, six experimental groups were established: (i) uninfected bees (Control), (ii) bees infected with *N. ceranae* (Infected), (iii) bees fed with syrup supplemented with 1 mM N‐acetylcysteine (NAC), (iv) bees infected with *N. ceranae* and fed with 1 mM NAC (INAC), (v) bees fed with syrup supplemented with 0.5 μg l^−1^ fipronil (PESTANAL^®^, Sigma‐Aldrich; FIP) and (vi) bees infected with *N. ceranae* and exposed to 0.5 μg l^−1^ fipronil (IFIP). Each experimental group was composed of 24 cages, each of which contained 45 bees (1080 bees per group) equally distributed from the five different colonies (i.e. nine bees from each colony per cage). Fipronil and N‐acetylcysteine treatments were administered 2 days before the infection that occurred 5 days after the emergence of bees (see below infection procedure). A stock solution of fipronil (70 g l^−1^) was prepared in DMSO, leading to a final percentage of 0.0025% DMSO in the feeding syrup. Therefore, caged honeybees from the Control, NAC, Infected and INAC groups were also fed with 0.0025% DMSO‐containing sucrose syrup. During the whole experiment, the honeybees were fed *ad libitum* with 50% sucrose syrup with 1% of Provita’ Bee (ATZ Dietetic), complemented or not with fipronil or NAC according to their experimental group. Every day, any dead bees were removed and scored, and sucrose consumption was quantified.

#### Midgut dissection and sampling method

Honeybees were anaesthetized with CO_2_ and euthanized by crushing of the thorax to ensure instantaneous death. The intestinal tract was dissected on a glass plate maintained on ice, and the first two‐thirds of the midgut was isolated and weighed before being immersed in liquid nitrogen and stored at −80°C. The sampling was performed at D0, five hours (H5) and 1, 2, 4, 7, 14 and 22 days after infection with *N. ceranae*. For each day, bees from three cages were euthanized for each of the six experimental conditions. Seven individual midguts/cage/condition/day were sampled for soluble peroxide measurement, four individual midguts/cage/condition/day for protein carbonylation and three pools of five midguts/cage/condition/day for measurement of malondialdehydes (a reliable marker of lipid oxidation). A minimum of five bees/cage/condition/day were conserved at −20°C for the counting of *N. ceranae* spores. The abdomen of each bee was dissected and homogenized in PBS using a manual tissue grinder. The parasite load was determined by counting with a haemocytometer chamber, and the Kruskal–Wallis test followed by Mann–Whitney analysis was used to compare the parasite load in the three infected groups.

#### Quantification of soluble peroxides in honeybee midguts

Midguts stored at −80°C were placed on ice and then crushed in 35 μl of catalase inhibitor (2 mg ml^−1^) and centrifuged at 15 000 *g* for 5 min. Thirty microlitres of the supernatant was stored at −80°C. For measurement, 10 μl of each sample was diluted to 1:5 and added to 950 μl of FOX reagent (sorbitol 100 mM; orange xylenol 100 μM; ferrous ammonium sulphate 250 μM; H_2_SO_4_ 25 mM) for 30 min of incubation at RT. To establish the standard curve, we prepared and treated a range of H_2_O_2_ concentrations in the same manner as the experimental samples. One hundred microlitres of each sample was loaded on a 96‐well plate in quadruplicate, and the plate was read at 596 nm using a microplate reader (Multiskan FC, Thermo Scientific).

#### Analysis of carbonylated proteins: preparation of the carbonylated BSA

A standard for carbonylation was prepared according to the protocol described by Yoo and Regnier (2004). Percentage of oxidation was controlled with standard carbonyl‐BSA (Cell Biolabs, Inc., San Diego, CA, USA). The oxidized BSA was frozen until used. Quantification of protein carbonyl groups is often used as an indicator of protein oxidation (Yoo and Regnier, 2004), and the relatively early formation and high stability of carbonylated proteins (Dalle‐donne *et al*., 2003) make this measure a suitable representation of global protein oxidation.

#### Analysis of carbonylated proteins: extraction and dosage of protein concentration

Midguts stored at −80°C were placed on ice and then crushed in 1 ml of RIPA buffer per 100 mg of tissue (RIPA buffer: Tris‐HCl 50 mM pH 8, NaCl 150 mM, DOC 0.5%, NP‐40 1%, SDS 0.1%) supplemented with PMSF 1 mM. The samples were incubated 15 min on ice and homogenized by vortexing for 5 min, and then centrifuged at 14 000 *g* for 15 min at 4°C. The supernatants were diluted to 1:40 for the Bradford assay and the rest was conserved at −80°C. The quantification of global proteins was performed following the protocol of the Coomassie Plus (Bradford) Assay Kit (Thermo Scientific). All samples were measured in triplicate at 596 nm.

#### Analysis of carbonylated proteins: protein derivation and slot blotting

The protocol was developed from previous studies (Robinson *et al*., 1999; Dalle‐donne *et al*., 2003) and adjusted for our study as follows: 50 μg of protein was incubated for 10 min at RT with SDS 20% at volume ratio of 1:0.5. One volume of 2,4‐dinitrophenylhydrazine (DNPH 5 mM final), diluted in HCL 2N, was added. The reaction was performed at RT for 30 min with homogenization every 10 min. One volume of neutralization buffer (Tris‐Base 2M; glycerol w/v 30%) was added. Three micrograms of protein samples and a range of carbonylated BSA quantities were slot‐blotted onto a polyvinylidene difluoride (PVDF) membrane using a slot blotter (PR 600 slot blot, Hoefer, Inc., Holliston, MA, USA). The membranes were treated with a primary anti‐DNP antibody (clone 9H8.1, Millipore™, Billerica, MA, USA) diluted at 1:2000 and then with a secondary antibody (anti‐mouse IgG HRP conjugate, Promega) diluted at 1:2500. Slot blot detection was performed using chemiluminescence (Clarity™ Western ECL Substrate, Bio‐Rad), and the readings were taken with an analyser (ChemiDoc™ MP System, Bio‐Rad, Hercules, CA, USA).

#### Lipid peroxidation in honeybee midguts

Lipid peroxidation (corresponding to the oxidation of unsaturated lipids) was titrated using the TCA method (TBARS Assay Kit, Cayman Chemical, Ann Arbor, Michigan, USA), which permitted us to measure malondialdehyde (MDA). The fluorometric version of the measure was conducted on a pool of three guts for each sample. Nine pools were measured in duplicate for each condition per day. The measurement of MDA with the TBARS method was considered representative of global lipid peroxidation.

### Statistical analysis

Statistical analysis was performed using stata software, version 13 (StataCorp, College Station, TX, USA) and r 3.2.5 software (https://cran.r-project.org/). The tests were two‐sided, with a type I error rate set at α = 0.05. Quantitative results were presented as the mean ± confidence interval (CI), according to statistical distribution (assumption of normality assessed using different tests: Shapiro–Wilk, Kolmogorov–Smirnov, Jarque–Bera and D'Agostino tests). Box–Cox transformation was applied to achieve normality when appropriate. Bee survival was analysed using the Kaplan–Meier method and compared using a Cox–Mantel test, and the success of *N. ceranae* development was estimated using a Kruskal–Wallis test followed by Dunn's test. For the results of consumption and analysis of the midgut samples, mixed models were used to take into account between‐ and within‐cage effect variability. Random‐effects models for correlated measures were used in place of usual statistical tests, which would be not appropriate because the independence of the data was not verified. Finally, principal component analysis (PCA) was considered to identify a set of latent constructs underlying some measured variables (oxidized lipids and proteins, and soluble peroxides). More precisely, we explored honeybee midguts over the course of the experimental timeline.

## Conflict of Interest

None declared.

## Supporting information


**Table S1.** *P*‐values obtained between different conditions with statistical analysis of consumptions and survival data for Exp.2.
**Table S2.** *P*‐values obtained between different conditions with statistical analysis for Exp. 2.
**Table S3.** Experimental days where a significant difference was observed between different conditions with statistical analysis for Exp. 2.
