# Supplementary material for: Disruption of oxidative balance in the gut of the western honeybee Apis mellifera exposed to the intracellular parasite Nosema ceranae and to the insecticide fipronil
Source: Microb Biotechnol. 2017 Jul 24;10(6):1702–17. doi: 10.1111/1751-7915.12772 (PMC5658624; doi:10.1111/1751-7915.12772)
Supplement: Supplementary file 1 — Table S1. P‐values obtained between different conditions with statistical analysis of consumptions and survival data for Exp.2. Table S2. P‐values obtained between different conditions with statistical analysis for Exp. 2. Table S3. Experimental days where a significant difference was observed between different conditions with statistical analysis for Exp. 2. [file MBT2-10-1702-s001.docx]

**Fig 1. Comparison of ROS concentration between *Nosema ceranae*-infected and uninfected honeybees.**

*

***

D0

H12

D1

D2

D4

D7

D17

0,0

0,2

0,4

0,6

0,8

1,0

1,2

1,4

Mean of fluorescence in honeybee midguts

(AU/mg +/- CI)

Time (Hour or Day post-infection)

Control

Infected

**Fig 2.** **Survival analysis of honeybees after infection by *Nosema ceranae* and/or exposure to the insecticide fipronil or to the antioxidant N-acetylcysteine.**

**Fig 3. Daily sucrose consumption curves of honeybees for the six experimental groups.**

0

10

20

30

40

50

60

70

80

90

100

D22

D14

D7

Time (Day post-infection)

Mean number of spores per honeybee abdomen

(x10

6

+/- CI)

Infected

INAC

IFIP

**Fig 4. Effect of the exposure to fipronil or N-acetylcysteine on N. ceranae spore production.**

**Fig 5. Principal component analysis of the three variables (lipid oxidation, soluble peroxide production and protein oxidation) in the honeybee midguts during the experiment timeline.**

**Fig 6. Lipid peroxidation quantification in the midguts for the six experimental groups.**

**Fig 7. Concentration of soluble peroxides in the midguts of honeybee exposed to fipronil or N-acetylcysteine and/or infected by N. ceranae.**

**Fig 8. Effect of the treatments on both protein concentration and protein oxidation.**

**Supporting Information**

**Table S1.** **P-values obtained between different conditions with statistical analysis of consumptions and survival data for Exp.2.** P-values were acquired when we compared two groups considering all combined days. For sucrose, N-acetylcysteine and fipronil, a mixed model analysis was applied on data, while for survival, Cox-Mantel test was used and finally, Kruskal-Wallis followed by Dunn test were chosen for the spore production. Ctrl: Control; Inf.: Infected.

| Treatment conditions | Sucrose 50% +  1% Provita’Bee consumption | N-acetylcysteine  1 mM consumption | Fipronil  0.5 µg/L  consumption | Honeybee survival | Spore production |
| --- | --- | --- | --- | --- | --- |
|  | p-values | p-values | p-values | p-values | p-values |
| Ctrl *vs* NAC | 0.121 | - | - | 0.136 | - |
| Ctrl *vs* FIP | **< 0.001** | - | - | **< 0.001** | - |
| Ctrl *vs* Inf. | 0.072 | - | - | **< 0.001** | - |
| Ctrl *vs* INAC | 0.362 | - | - | **< 0.001** | - |
| Ctrl *vs* IFIP | 0.189 | - | - | **< 0.001** | - |
| NAC *vs* FIP | 0.194 | - | - | **< 0.001** | - |
| NAC *vs* Inf. | 0.996 | - | - | **< 0.001** | - |
| NAC *vs* INAC | 0.948 | 0.446 | - | **< 0.001** | - |
| NAC *vs* IFIP | 0.999 | - | - | **< 0.001** | - |
| FIP *vs* Inf. | 0.249 | - | - | **< 0.001** | - |
| FIP *vs* INAC | **0.029** | - | - | **< 0.001** | - |
| FIP *vs* IFIP | 0.086 | - | 0.065 | **< 0.001** | - |
| Inf. *vs* INAC | 0.608 | - | - | 0.100 | 0.399 |
| Inf. *vs* IFIP | 0.860 | - | - | 0.819 | 0.469 |
| INAC *vs* IFIP | 0.688 | - | - | 0.959 | 0.919 |

**Table S2.** **P-values obtained between different conditions with statistical analysis for Exp. 2.** P-values were acquired when we compared two groups considering all combined days for each of the four measures with the application of a statistical mixed model. Ctrl: Control; Inf.: Infected.

| Treatment conditions | Soluble peroxides | Lipid  peroxidation | Total  proteins | | Protein  carbonylation | |
| --- | --- | --- | --- | --- | --- | --- |
|  | p-values | p-values | p-values | | p-values | |
| Ctrl *vs* NAC | **0.002** | 0.716 | 0.666 | 0.603 | |  |
| Ctrl *vs* FIP | 0.252 | 0.309 | 0.385 | 0.134 | |  |
| Ctrl *vs* Inf. | **0.023** | 0.298 | **0.006** | **< 0.001** | |  |
| Ctrl *vs* INAC | **< 0.001** | 0.113 | **0.006** | **0.001** | |  |
| Ctrl *vs* IFIP | **0.003** | 0.255 | 0.386 | **0.002** | |  |
| NAC *vs* FIP | **0.001** | 0.944 | 0.987 | 0.797 | |  |
| NAC *vs* Inf. | **< 0.001** | 0.502 | 0.083 | **< 0.001** | |  |
| NAC *vs* INAC | **< 0.001** | 0.189 | 0.079 | **0.006** | |  |
| NAC *vs* IFIP | **< 0.001** | 0.434 | 0.987 | **0.034** | |  |
| FIP *vs* Inf. | 0.592 | 0.114 | 0.175 | **< 0.001** | |  |
| FIP *vs* INAC | **0.002** | **0.027** | 0.168 | **< 0.001** | |  |
| FIP *vs* IFIP | 0.196 | 0.090 | 1.000 | 0.269 | |  |
| Inf. *vs* INAC | **0.037** | 0.829 | 0.999 | 0.699 | |  |
| Inf. *vs* IFIP | 0.748 | 0.994 | 0.122 | **< 0.001** | |  |
| INAC *vs* IFIP | 0.093 | 0.655 | 0.060 | **< 0.001** | |  |

**Table S3. Experimental days where a significant difference was observed between different conditions with statistical analysis for Exp. 2.** P-values were acquired when we compared two groups considering each day taken independently for each of the four measures with the application of Kruskal-Wallis followed by Dunn tests. /: no difference. Ctrl: Control; Inf.: Infected.

| Treatment conditions | Soluble  peroxides | Lipid peroxidation | Total  proteins | | Protein carbonylation | |
| --- | --- | --- | --- | --- | --- | --- |
| Ctrl *vs* NAC | 1 to 22 | 1 | | 4, 14, 22 | H5, 4, 7, 22 |  |
| Ctrl *vs* FIP | 0, H5, 4, 14 | 4 | | 0, H5, 4 | 0, H5, 2 |  |
| Ctrl *vs* Inf. | 7, 22 | 1,4,14 | | 1, 4, 14, 22 | 1, 2, 22 |  |
| Ctrl *vs* INAC | H5, 1, 4, 7 | 2,4,7,14 | | H5, 1, 4, 14, 22 | 1, 14, 22 |  |
| Ctrl *vs* IFIP | H5, 1, 22 | 1,2,4,7,14 | | 1, 4, 7, 14, 22 | H5, 1, 4, 14 |  |
| NAC *vs* FIP | H5,1, 2, 4, 22 | / | | 0, 4, 14 | 4, 22 |  |
| NAC *vs* Inf. | H5, 1, 2, 7, 14, 22 | 14 | | 1, 4 | H5, 2, 4, 7 |  |
| NAC *vs* INAC | H5, 2, 7, 14, 22 | 1, 2, 7, 14 | | 1, 4 | 1, 4, 7 |  |
| NAC *vs* IFIP | H5, 4, 7, 14, 22 | 1, 7, 14 | | 1, 4 | 4, 14, 22 |  |
| FIP *vs* Inf. | H5, 4, 7, 14 | 14 | | 1, 7, 14 | H5, 2, 7, 22 |  |
| FIP *vs* INAC | 1, 4, 7, 14 | 2, 7, 14 | | 14 | 1, 2, 4, 7, 22 |  |
| FIP *vs* IFIP | H5, 1, 2, 4, 14, 22 | 1, 2, 7, 14 | | 1, 14 | 4, 14 |  |
| Inf. *vs* INAC | 1 | 1, 7 | | / | 2 |  |
| Inf. *vs* IFIP | H5, 1, 7 | 1, 7 | | 7 | H5, 2, 4, 7, 22 |  |
| INAC *vs* IFIP | H5, 1, 2, 4, 7 | H5 | | / | 1, 4, 7, 14, 22 |  |
